# Web-based single session therapy training for mental health support providers: a mixed-methods evaluation study protocol

**DOI:** 10.1007/s44192-024-00122-0

**Published:** 2024-12-30

**Authors:** Jasmine Joseph, Santhosh Kareepadath Rajan, N. T. Sudhesh, Uma Krishnan

**Affiliations:** School of Psychological Sciences, Christ University, Bangalore, 560029 India

**Keywords:** Single session therapy, Training, Evaluation study design, Mixed method, Randomized control design

## Abstract

The growing mental health needs and constrained resources in low- and middle-income countries necessitate scalable solutions. Single Session Therapy (SST) is a global trend in brief and cost-effective options for mental health interventions. It involves a single planned session between mental health service provider and client. This study aims to present a protocol to develop and evaluate a culture specific web-based training program to equip mental health support providers with the skills and confidence to deliver SST. The study protocol uses a mixed-methods evaluation design through three phases—need assessment – where psychologists and social workers collaborate to identify training needs and co-create the program; development and expert validation of the web-based training program; and randomized control trial to evaluate the training, followed by in-depth discussions with participants. This study breaks new ground by empirically designing and evaluating a training program for SST. It uniquely co-designs and validates a culturally sensitive SST training program, leveraging the expertise of a renowned international panel. This protocol goes beyond a blueprint for replicating this study, it serves as a foundational guide for nations seeking to implement effective SST training for their mental health professionals, preventing duplication of efforts.

**Supplementary Information:**

The online version contains supplementary material available at 10.1007/s44192-024-00122-0.

## Introduction

Single Session Therapy (SST) is a well-established form of psychotherapy with origins dating back to the early days of psychotherapy. Having been practiced and researched for over thirty years [1], SST has gained significant traction in recent years due to its potential, efficacy and cost-effectiveness. SST is defined as a brief encounter where both the therapist and client anticipate that it may involve only one session [2, 3]. SST originates from the claim that the most frequent number of therapeutic sessions experienced by clients globally is ‘one’, and more than 50% of these sessions are reported to be satisfactory [3–5]. SST is appreciated for its accessibility when needed and its alignment with clients' overall experiences in the context of psychotherapy [6]. SST takes a pragmatic approach by focusing on the client's immediate concerns and engaging in collaborative exploration of potential solutions. It has a potential to help ease the mental health crisis by providing evidence-based, low-intensity, and shorter-duration treatments, overcoming time constraints and accessibility barriers linked to traditional therapy models [7]. The focussed approach of SST necessitates therapists to play a crucial role in reframing the client's concern, bolstering their coping skills, and motivating them to apply the insights gained from the session to initiate meaningful change [8]. Developing a 'single session mindset' in therapists through targeted training is crucial for optimizing the outcomes of SST interventions. Researchers from other low- and middle-income countries (LMIC) can leverage this study protocol designed for Indian mental health support providers, adapting it to deliver culturally appropriate SST training in their countries.

American Psychological Association [9] released their third Practitioner Pulse Survey data in December 2023, highlighting the increasing demand for mental health support and the lack of availability of providers in the United States. Similar mental health burden and the challenges of providing adequate care have been documented in India [10]. Substantial number of individuals (13.67%) in India grapple with mental health issues, with a significant proportion (84.5%) not actively seeking support, and many facing challenges in accessing the necessary treatment [11, 12]. The limited availability of mental health practitioners, resulting from both workforce shortages and individual schedule constraints, hinders their ability to adequately serve all individuals seeking help [11, 13]. To address the challenges faced in improving mental health services in India, scalable interventions leveraging technology and innovative approaches need to emerge [14, 15].

Online single-session therapy can be a viable and acceptable option for individuals whose mental health needs may not be getting adequately addressed especially in LMICs [16–18]. Despite the extensive research indicating that ‘one’ is the most common number of therapy sessions attended by clients worldwide, apprehensions about this form of service delivery from existing practitioners is to be expected [19]. Dedicated books and numerous articles, exist solely to address the ever-present questions (FAQs) surrounding SST [20–22]. Empirical studies from India also concluded that attitudes constitute a critical barrier to the implementation of brief interventions and modifying these attitudes through targeted trainings can yield significant improvements in healthcare practices [23].

### Theoretical framework

The study is grounded in the Theory of Planned Behavior (TPB) proposed by Ajzen in 1985 [24]. The TPB posits that attitudes, subjective norms, and perceived behavioural control, closely linked to self-efficacy, predict individuals' intentions and ultimately their behavior. The training program aims to improve attitudes towards SST by highlighting its benefits and evidence-based practices. Additionally, addressing cultural norms and involving experts can reinforce subjective norms favoring SST. Finally, the design of the training and its skill-building exercises can enhance participants' perceived ability or self-efficacy to deliver effective support in a single session format. The current study measures attitude, beliefs and self efficacy as primary variables.

### Operationalization of key concepts

Attitude and self-efficacy are frequent subjects of research, especially as measures of outcome of clinical training program for mental health support providers [25–27]. The most suitable attitude scale for single session therapy is the Belief and Attitude towards Therapy Questionnaire (BAT-Q) developed by Bolter in 1987 [28] as part of her thesis. This scale measures the attitude and beliefs of therapists towards brief psychotherapy, one of the founding members of SST, Michael Hoyt was part of this study. This scale was last used to investigate the impact of training on therapists' acceptance of brief therapy, as the primary measure of positive attitude shifts [29].

Karaırmak [30] underlines the potential of a high-quality training program to elevate therapist’s sense of self- efficacy. The Counseling Self-Estimate Inventory (COSE) is a widely used scale to measure factors that reflect counselor trainees' self-efficacy and confidence in using microskills, attending to process, dealing with difficult client behaviours, behaving in a culturally competent way, and being aware of one's values [31]. In a study with Turkish therapists Karaırmak [30] proposed a modified version of the scale, COSE-TR, arguing for its enhanced suitability for collectivist cultural contexts.

### Rationale of the study

SST demands swift and accurate assessment of client needs and immediate formulation of effective interventions within a single session. Therapists trained in traditional models might experience apprehensions about the limited time and potential for insufficient intervention in SST, impacting their confidence and delivery. The absence of readily available training resources in LMIC restricts access to qualified SST practitioners. This hinders broader implementation of SST and inhibits advancements in the field and its potential to contribute to the overall evolution of mental health care. Developing a suitable web-based training program and researching mental health support providers’ attitudes towards SST will provide crucial insights into their willingness to adopt this model and potential areas of resistance that need to be addressed.

Despite a global presence of SST training programs, including those offered by LaTrobe University, Australia and the University of Toronto, Canada, and even programs led by individual experts, a critical research gap exists in terms of empirically validating the effectiveness of any SST training program. The application of SST in culturally diverse contexts like India would add to the body of knowledge on SST. Indian universities currently lack academic offerings focused on single-session interventions, highlighting a critical gap in formal training and knowledge dissemination. This absence further underscores the need for culturally tailored SST training programs specifically designed for Indian mental health support providers. The outcomes of this research protocol will pave the way for broader SST adoption and improved mental healthcare delivery in India.

### Aim of the study

This study aims to develop and empirically validate a culturally-tailored, web-based SST training program specifically designed to improve the attitudes, values, and self-efficacy of mental health support providers in delivering effective SST interventions.

### Research objectives

1. To explore the alignment of the proposed training curriculum and perceived training needs of mental health support providers regarding Single Session Therapy (SST).
2. To transform and validate a web-based version of the SST training program, ensuring content validity through expert review.
3. To assess the effect of the SST training program in improving the attitudes, values, and self-efficacy of mental health support providers related to delivering single-session therapy interventions.

### Research questions

1. What successful features and design elements found in the literature can be adapted to maximize engagement and learning within an online training program for mental health support in Indian cultural context?
2. What is the level of agreement among experts on the content of each module in the web-based SST training program to be accurate and aligned with evidence-based practices in Single-Session Therapy?
3. Does the online SST training program significantly improve mental health support providers' attitudes and beliefs towards SST compared to a waitlist control group?
4. Does the online SST training program significantly increase mental health support providers' counseling self-efficacy in delivering SST compared to a waitlist control group?
5. What are the experiences of participants who show the most significant and least significant changes in attitudes, beliefs and self-efficacy towards SST?

## Methods

This study protocol follows the Standard Protocol Items: Recommendations for Interventional Trials (SPIRIT) 2013 statement with the latest outcome-specific items as of 2022 [32]. The checklist is attached as Supplementary appendix.

### Study design

The multi-faceted nature of this research investigating the development and evaluation of a web-based SST training program necessitates a mixed-methods evaluation design, as advocated by Creswell and Clark in 2018 [33]. Such multi-phase design allows to delve into the intricacies of program construction and assessment, validation, and effectiveness check. This research design is commonly used for program evaluations, where a combination of core (quantitative, qualitative and mixed method) designs is applied to facilitate the development, adaptation, and assessment of particular programs [33].

The selection of a mixed-methods evaluation design for this research aligns with the multi-faceted nature of the objectives [34]. The choice of this complex design is appropriate when the research interest is to evaluate the process of construction of an online training program and assess the outcomes [33, 35]. Mixed-methods evaluation design has been employed to design and assess other web-based mental health support training programs [36].

**Fig. 1:**
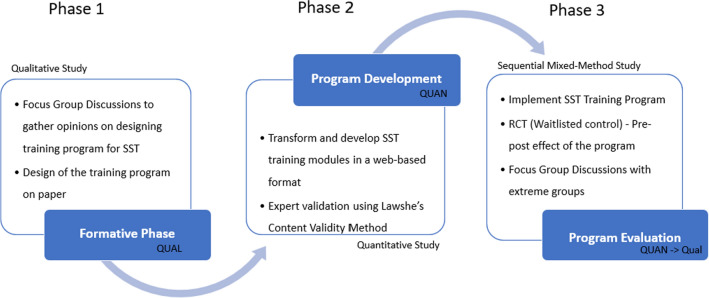
Research design

This design follows a pragmatic approach and involves distinct stages of needs assessment to identify program goals, process evaluation to monitor implementation, and outcome evaluation to assess the program's impact as explained in Fig. 1.

The initial phase, constituting the needs assessment and formative stage will establish the groundwork. A review of the literature on creating online training programs for psychology or social work students of India, will serve as the basis of the development of the program. Focus group discussions (FGD) with mental health support providers will validate and enrich the findings. Incorporating these findings, established SST training frameworks, and the researcher's expertise, the initial phase will involve the conceptualization of an SST training program on paper. The second phase will transform this design to a web-based training program. To ensure content validity, the program will undergo a rigorous review by SST experts using Lawshe's Content Validity method. The third phase, will administer the training program to participants in a randomized control design, facilitating the measurement of changes in participants' attitudes, beliefs, and self-efficacy related to delivering SST. For an in-depth understanding of the quantitative outcomes, qualitative data is collected through focus group discussions among two groups – highest and lowest variations in attitude and self-efficacy. In the three-phase design, each phase is directly linked to a corresponding objective of the research.

### Phase 1 – formative phase – focus group discussions

**Fig. 2:**
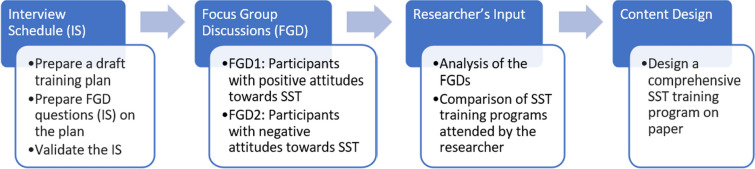
Formative Phase – Focus Group Discussions

The formative phase employs a qualitative study for the design of the SST training program. Figure 2 shows the various steps involved.

#### Participants

Two focus group discussions are planned, each comprising 5 participants. The first group will consist of individuals who show a positive attitude towards single-session interventions during the recruitment process, while the second group will include participants with a negative stance. Sampling design is purposive sampling.

##### Inclusion criteria

- mental health support providers in second year or with a completed Master's degree in psychology or social work
- awareness of at least one approach towards psychotherapy
- attendance of a minimum of two online self-guided training program on psychotherapy in the past

##### Exclusion criteria

- diagnosis of mental health issues
- uncomfortable communicating in English language
- attended any training in single session therapy in the past
- planning or enrolled to attend any other related trainings during the period till the FGDs are conducted

#### Procedure

The recruitment flyers will be prepared inviting participants for the two groups. The flyers will be disseminated through professional organizations, university psychology/social work departments, and online forums for mental health professionals. The participants invited for FGD will be required to provide written informed consent. Separate FGDs will be conducted in English with each group and voice recordings will be collected. A semi-structured interview guide is developed and validated by experts. All questions are based on the Initial training plan of the program (Table 1). The design of the training program is based on the training published by The Bouverie Centre, La Trobe University, Australia as “Putting Single Session Thinking to Work” [37]. It is also informed by the first author’s participation in various professional development activities. These include—A 6-h online workshop on single-session therapy led by Prof. Windy Dryden, a 7-h self-guided continuing professional development (CPD) course titled "Single Session Thinking" offered by La Trobe University, Australia, a 4-h pre-recorded online lecture video by Monte Bobele on "Introducing Single-Session/Walk-in Therapy," provided by the American Psychological Association (APA), a 12-h online instructor-led course on "Solution Focused Single Session Therapy" from the University of Toronto, Canada, and a 15-h live online course on "Single Session Therapy Skills Training" by Gauri Row Kavi from Self Align, India.

**Table 1:** Initial training program plan draft

Module Title	Learning Objectives	Digital Pedagogy	Type	Time (est.)
Introduction to Single Session Therapy (SST)	* Define single session therapy (SST) * Explain the rationale * Theoretical reasoning of using SST * FAQs about SST	* Video lectures * Infographics * Self-reflection exercises * Discussion Forums	Mandatory	1 Hrs
The SST Framework	* Describe the key stages of the SST framework * Explain the application of each stage in a single session * Identify ethical considerations in applying SST * Do's and Dont's in SST	* Infographics * Interactive presentations * Animated diagrams	Mandatory	1 Hrs
Therapeutic Skills for SST	* Demonstrate various SST skills * Formulate open-ended questions to facilitate client exploration * Establish rapport and build a therapeutic alliance within a single session * Intervention techniques from various therapeutic approaches * Individualized intervention plans for diverse client presentations (through case study analysis)	* Video demonstrations * Interactive simulations (text chat) * Case studies (text & video) * Self guided—Question and Answer	Mandatory	4 Hrs
Adaptations and Cultural Considerations in SST	* Indian adaptations in SST * Cultural biases and strategies to mitigate them * Develop culturally sensitive communication skills for SST	* Case studies (text based interactive) * Youtube links * Clinical decision-making exercises	Optional	10 h
Effectiveness research on SST	* Evolution of Single session mindset—History * Research Synthesis * Different therapeutic approaches in single session format	* Infographics * Links to research * Resource Library	Optional	10 h
Case Studies and Practice Exercises	* Analyze and apply SST principles to real-world scenarios * Practice and refine therapeutic skills in a simulated environment	* Interactive case studies (video based) * Simulated role-playing exercises * Peer-feedback forums	Optional	10 h

#### Measures

As shown in Fig. 1, an interview schedule will be developed based on the findings of the literature review and based on the initial draft training program. This will be validated by three experts, preferably experienced in online psychotherapy training and qualitative research. These questions will explore participants' perceptions of the draft online training on SST, and their preferences for the program design. The validated interview schedule will be included in a PowerPoint presentation, alongside the draft training plan. The FGDs with both the positive and negative groups can be conducted either online or offline, with the training plan projected during the discussions.

#### Data analysis

Reflexive thematic analysis by Braun and Clarke [38] using an inductive approach will be employed for both the transcribed FGD recordings. This approach will facilitate the identification of key themes and patterns across existing knowledge and stakeholder perspectives. These insights will guide the revision of the training plan and incorporation of additional content and features to the draft plan. Member checking will be conducted on the transcriptions, the analysis and the revised training plan to enhance the credibility and validity of the study. This phase will culminate in the development of a culturally tailored SST training program, drawing upon both research findings and the researcher’s prior experience.

### Phase 2—program transformation and validation

**Fig. 3:**
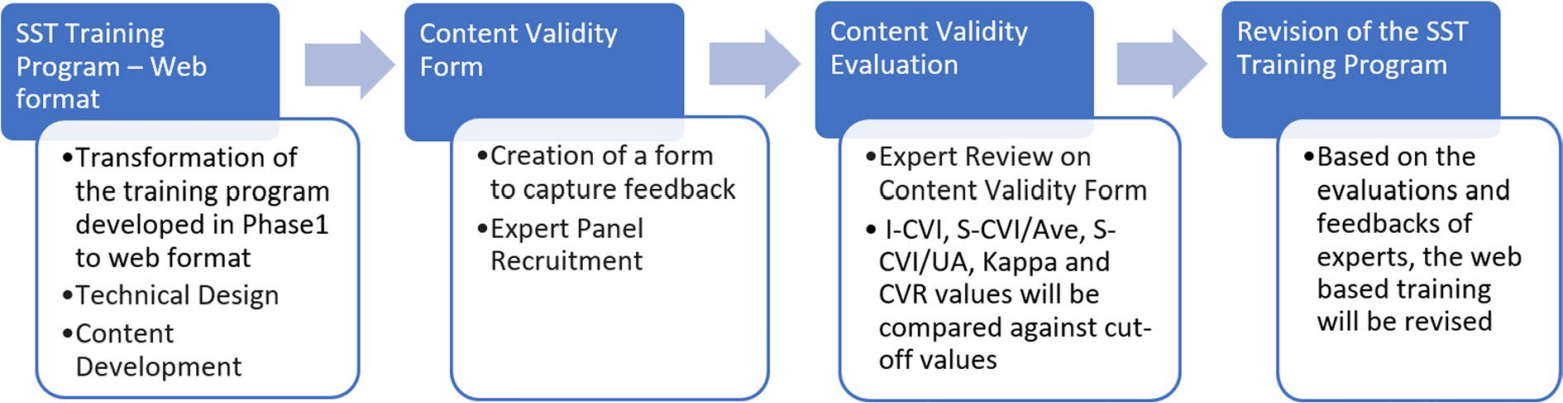
Transformation and Validation of the Web-based Program

The online training development phase will utilize the data collected in Phase 1 to build a web-based SST training program, either self-hosted or on any other learning platforms. Each module will have multiple chapters according to the learning objectives as given in Table 1. A quantitative content validity assessment will be conducted to evaluate the program. Figure 3 shows the various steps involved.

#### Participants

The content of the web application will undergo a rigorous evaluation by a panel of ten experts, drawn from both India and abroad in accordance with the guidance provided by Ayre and Scally [39]. The inclusion criteria are mental health support providers with at least five years of experience in practicing SST or experience in providing training or supervision on psychotherapy. Purposive sampling is used to request experienced SST practitioners in the researcher’s social network. A list of influential authors on SST can be obtained from a bibliometric study on SST [40], and contacts can be obtained from the professional networking site of LinkedIn.

#### Procedure

Potential expert participants will be recruited through email. They will receive a document with the study description, informed consent form, the link to the training website and the corresponding content validity form for each module of the training. Experts will be encouraged to provide detailed written comments and suggestions for improvement, focusing on content accuracy, comprehensiveness, and relevance to learning objectives. The program will then be revised based on both the quantitative data from the content validity assessments and the qualitative insights from the experts' written feedback.

#### Measures

A content validity form is developed specifically for this study. This form will gather feedback on three domains for each chapter in the training program—Content accuracy, Content comprehensiveness and User-friendliness. Each expert will independently rate each chapter on these three domains using a 4-point Likert scale (1 = strongly disagree, 4 = strongly agree) and fill the content validity form electronically. All data will be collected, anonymized, and stored securely.

#### Data analysis

Similar to the Lawshe's method, item-level content validity index (I-CVI) will be calculated for each chapter by dividing the number of experts who rate it as 3 or 4 by the total number of experts. Scale-level content validity index (S-CVI) is calculated using two methods for each module. S-CVI/Ave—the average of all I-CVI scores for all items in each module. S-CVI/UA (Universal Agreement)—the proportion of items in each section that receive an I-CVI of 3 or 4 by all experts. Kappa statistics to assess the level of agreement among experts will also be calculated. Cut-off values for acceptable content validity is: I-CVI ≥ 0.78, S-CVI/Ave ≥ 0.90, S-CVI/UA ≥ 0.80 and Kappa ≥ 0.74 [41]. Written comments and suggestions provided by experts will be utilized to identify potential areas for improvement in the program.

### Phase 3 – program evaluation—RCT

**Fig. 4:**
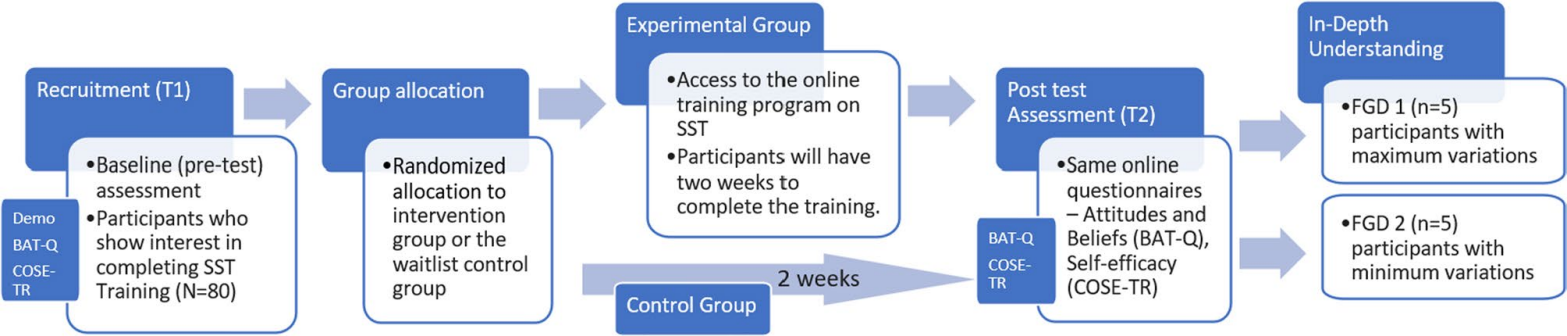
Program Evaluation – Randomized Control Trial

The efficacy of the SST training program developed and validated in previous phases will be evaluated using a parallel group sequential mixed methods randomized control design with a waitlist control group. Figure 4 shows the various steps involved.

#### Participants

Mental health support providers (e.g., psychologists, counsellors, social workers) from India, who express interest in completing the SST training program will be invited to participate. Eligibility criteria will require participant to be either in their final year or have completed a Master's program in psychology or social work from India. They should commit availability to participate in the 2 week long study. Participants who have received SST training in the past are excluded. Those currently in any treatment for mental illness will also be excluded. Age and gender will not be not a criterion for exclusion. The quantitative component of this phase will require a large sample size. Potential recruitment strategies include reaching out to universities offering mental health related master's level programs, training institutes offering CPD trainings, collaborating with country-specific mental health societies and organizations, utilizing social media platforms or online forums, targeted email invitations and employing snowball sampling techniques. Offering incentives like certificates of completion can motivate potential participants.

#### Sampling design

Sampling involves purposive sampling for recruitment and then random assigned to experimental and waitlist control groups in 1:1 ratio. To ensure allocation concealment, an independent researcher, unaffiliated with this study, will be responsible for generating the randomization sequence. This researcher will not be involved in participant recruitment, enrollment, or data collection. Randomization can be achieved using the research randomizer software [42]. Participants will be blinded to their group assignment throughout the study. Unblinding will only be permitted under exceptional circumstances and with prior approval from the research ethics committee.

The sample size for this study is determined to be 80 participants, considering an anticipated 25% dropout rate. This decision is informed by the considerations outlined in a similar randomized control trial (RCT) to study the efficacy of a skills training program for 58 mental health support providers by Perlman et al. [43]. The sample size of 60 participants will provide 80% power to detect an effect size of 0.8 [44] between the intervention group and the control group at 5% level of significance (two-tailed test) as evidenced in a similar study by Ho et al. [45] with 68 participants.

To explore contrasting perspectives and for an in-depth understanding of the participant’s experiences, focus groups will be chosen using a targeted homogeneity sampling method, aiming to include those with both the highest and lowest score variances on attitude and self-efficacy scales.

#### Operational definitions

**Single session therapy.** Neff et al. [29] acknowledged that defining brief therapy is difficult and that it embodies a distinct mindset for both therapist and client, often driven by resource limitations. It could be thought of as therapy where time is allotted and treatment is rationed, emphasizing deliberate brevity over coincidental shortness. The proposed operationalization aligns precisely with the core principles of SST [2].

**Attitude towards SST.** Attitudes are described as positive or negative affective responses towards a specific psychological concept. For the BAT-Q tool [28] the attitude scale measures the attitudes of therapists towards brief therapy.

**Beliefs about SST.** Beliefs or values in the context are seen as underlying beliefs about the best way to conduct psychological interventions. For the BAT-Q tool [28] the value scale measures the perceived worth, significance, or importance placed on brief therapy by the therapists.

**Counseling self-efficacy.** Karaırmak [30] defined counseling self-efficacy as a counselor's confidence in their ability to skilfully orchestrate and utilize various techniques when working with clients in the future. The COSE-TR scale defines four factors. ‘Microskills’ factor in the COSE-TR tool operationalizes counselors' self-efficacy by assessing their confidence in adeptly utilizing various counseling techniques. The ‘Dealing with Difficult Client Behaviors’ factor evaluates counselors' self-efficacy in their fundamental knowledge, confidence in using open- or closed-ended probes, and addressing difficult client responses. ‘Cultural Competence’ measures counselors' self-efficacy in demonstrating an understanding and effectiveness in working with clients from diverse backgrounds, including their ability to relate to clients of different socioeconomic status and view situations from various cultural perspectives. The ‘Counseling Process’ factor assesses counselors' self-efficacy in guiding the therapeutic journey, including their worries about interpretation and confrontational responses, uncertainties in leading clients toward goal development, and concerns about facilitating client progress throughout the counseling relationship.

#### Measures

The quantitative data collection for both pre-test (T1) and post-test (T2) time points (before and after completing the online SST training) will utilize the same instruments BAT-Q and COSE-TR. All data will be collected online using google form.

The Beliefs and Attitudes Toward Therapy Questionnaire (BAT-Q) developed by Bolter [28], measures therapists' preferences for long-term versus short-term therapy approaches. Comprising 19 items, the BAT-Q divides into "values" and "attitudes" subscales, resulting in a total score. While the "values" subscale relies on 13 items, the "attitudes" subscale relies on the remaining six. It utilized a seven-point Likert scale and some of the items are reverse scored. A higher subscale and total score suggest higher inclination towards brief therapy. The BAT-Q established itself reliable with Cronbach alpha as 0.88 and acceptable item and test–retest reliability [29, 46]. However, its terminology requires updating for use in the context of SST. The study will adapt BAT-Q to the SST domain by replacing terms like "brief therapy" with "single-session therapy" and "patients" with "clients".

The Turkish version of Counseling Self-Estimate Inventory (COSE-TR) [30] is a shorter version of the original COSE scale [31] with 27 items designed for measuring counseling self-efficacy in collectivists culture. It demonstrates strong internal consistency, with a Cronbach's alpha coefficient of 0.90 and, the four identified factors within the scale display satisfactory reliabilities: Microskills (0.90), Dealing with difficult behaviors (0.71), Cultural competence (0.71), and Counseling process (0.79). The total score represents the overall performance by summing the scores of all individual items across all factors.

To gain a deeper understanding of the factors influencing changes in participants' attitudes, values, and self-efficacy, focus group discussions will be conducted with a purposive sample of participants who exhibit the largest and smallest pre-post test score differences. For participants with large positive score differences, the questions will focus on the specific aspects of the training program that were most helpful. For participants with large negative score differences, the questions will focus on the aspects of the training program that were less effective. The interview schedule will be validated by qualitative research experts.

#### Intervention

The initial training plan outlined in Table 1 will serve as a foundation for the development of the final web-based training program. This plan will undergo rigorous revisions in phases 1 and 2 of the research. The finalized web-based training program is delivered as the intervention to participants in this phase 3 of the research. The final program will consist of both mandatory and optional components. This phase will involve a pre-post test administered to participants who have completed at least the mandatory training hours within a two-week timeframe. To promote adherence to the training time duration, participant progress will be closely monitored, and email reminders will be sent every two days. The waitlisted control group drawn from the same pool as the intervention group, helps mitigate selection bias. Control group will be offered the training program after post-test data is collected from both the groups post two weeks.

#### Procedure

Participants will be recruited using personalized social media messages, targeted social media advertisements, email outreach, flyers within relevant settings, and leveraging the researchers’ professional network. Demographic data as well as their interest in completing in SST training, will be collected through a researcher-developed questionnaire. Participants who show interest in completing the SST training will be randomly allocated to either the intervention group or the control group using the research randomizer software program in 1:1 ratio. The intervention group will have access to the online training program immediately for a period of two weeks. The waitlisted control group will not have access to the online training program during the intervention period, and are offered access after post-test at the end of two weeks.

The pre-test data (T1) is collected using two scales. The first one is a modified version of the BAT-Q Scale [28] with replaced terms like "brief therapy" with "single-session therapy" and "patients" with "clients". These revisions in the item texts of the tool will undergo validation by three psychometric experts. Given that the changes are minor and primarily involve replacing terms to ensure clarity and relevance to the specific context of SST, a consensus among two experts can be considered a reliable validation. The second scale is the counselling self-efficacy scale, COSE-TR [30].

At the end of the two weeks, participants in both the intervention group and the control group will complete a post-test assessment (T2). Participants demonstrating the most significant and least significant changes in attitude and self-efficacy between T1 and T2 will be invited to two separate 30-min focus groups (top 10 and bottom 10, expecting a 50% response rate). This targeted approach facilitates the exploration of contrasting experiences. The SPIRIT Figure in Table 2 explains the schedule of enrolment, interventions, and assessments.

**Table 2:** SPIRIT Figure for the Schedule of Enrolment, Interventions, and Assessments

Study period					
	Enrolment	Allocation	Post-allocation		Close-out
Timepoint	T_1_	0	Week 1	Week 2	T_2_
ENROLMENT					
Eligibility screen	X				
Informed consent	X				
Random Allocation		X			
INTERVENTIONS					
SST Training Group			Two weeks access to training		
Waitlist Control Group			No access to training		Two weeks access to training after assessments
ASSESSMENTS					
Quantitative					
BAT-Q	X				X
COSE-TR	X				X
Qualitative					
Selected participants					X

#### Data analysis

Descriptive statistics will be provided for baseline (T1) data. Participant demographics (e.g., age, gender, profession, years of experience, setting) will be described using frequencies and measures of central tendency as appropriate. Internal consistency (e.g., Cronbach's alpha) of both BAT-Q and COSE-TR for Indian population will be evaluated using Jamovi 2.4 software. Missing data will be handled with multiple implementations. Normality of the data will be assessed using the Shapiro–Wilk test.

If the data is found to be normally distributed, independent samples t-test will be used to report the baseline characteristics of the intervention and control groups. ANCOVA will be used to compare the post-intervention outcomes (T2) (self-efficacy, values and attitudes) between the intervention and control groups while statistically controlling for baseline (T1) differences. If the ANCOVA reveals a significant group effect, post-hoc comparisons will be conducted to identify specific differences. Paired samples t-test will be used to analyze changes within each group from pre-test (T1) to post-test (T2) to assess whether there are significant changes over time within each group. If the data is not normally distributed, Templeton’s two-step approach for transformation will be applied. After transformations if the data fails to meet the normality assumption, Mann–Whitney U test will be used to report baseline characteristics. Kruskal–Wallis test can be to identify specific differences between groups. Wilcoxon Signed-Rank Test can be used to assess significant changes over time within each group. All changes will be reported for attitudes, values and four subdomains of self efficacy and p values below 0.05 will be considered significant. Jamovi software will be used for statistical analysis.

To understand the factors influencing the changes observed in the quantitative data, a qualitative analysis will be conducted using six step thematic analysis described Braun and Clarke [38]. Focus group recordings will be transcribed verbatim. The transcripts will be systematically coded, identifying recurring ideas, patterns, and significant quotes related to participants' experiences and explanations for their attitude and self-efficacy changes. Thematic comparisons will be drawn between participants who exhibited significant positive and negative changes in their attitudes, values, and self-efficacy.

## Ethical considerations

The Institution Review Board of the authors’ University has provided ethical clearance (CU: RCEC/00583/02/24) for the research proposal. Throughout the research, adherence to the APA codes of ethics is maintained. Participants are required to provide informed consent, ensuring they have a clear understanding of the purpose, procedures, and their right to withdraw at any time. Voluntary participation is emphasized, with no negative consequences for non-participation or withdrawal. Confidentiality and privacy of participants' personal information is protected through secure data storage and anonymization, with access to the researchers only. Participation in this study is expected to be safe and without any foreseen negative consequences. As such, no specific guidelines for stopping participants early are needed, and no interim analyses are planned. This publication is based on protocol version 1 (02–03-2024). Protocol modifications will be reported in forthcoming publications, if applicable. The research findings will be disseminated through publications in peer-reviewed journals, presentations at relevant research conferences, and other appropriate platforms. Authorship for all publications will follow the Vancouver recommendations for ethical authorship. Recruitment for the study is expected to be completed by February 2025.

## Expected outcomes

The first phase of needs assessment and curriculum development explores the alignment between the proposed SST training curriculum and the perceived training needs of mental health support providers. The findings from this phase will be used to develop a detailed training curriculum that addresses the identified needs. Second phase involves the development and validation of a web-based SST training program to ensure its content validity and alignment with evidence-based practices. It is hypothesised that there will be a high level of agreement among experts on the content validity of each module in the web-based SST training program. The final phase of the study will involve a randomized controlled trial to evaluate the effectiveness of the online SST training program. The hypothesis is that the intervention group will demonstrate significantly greater improvements in attitudes, values, and self-efficacy towards SST compared to the control group. The qualitative data from the FGDs in this phase will explain the factors that influenced their changes.

## Implications of the research

This research offers potential contributions to society, mental health support providers, and research. For the society, SST training for mental health support providers could expand the availability of brief, cost-effective therapy, making it more accessible for individuals who might face limited time or financial resources. By promoting awareness and understanding of SST as a viable option, the research could contribute to destigmatizing mental health interventions and encourage help-seeking behavior.

For mental health support providers, it could enhance the skills and confidence, potentially leading to better client outcomes. Effective SST training could enhance job satisfaction by allowing them to efficiently help more clients and potentially reduce burnout rates in the mental health field. While SST has gained traction and research attention in developed nations like Australia, the United States, the United Kingdom, Canada, and Sweden, its potential impact may be even greater in LMICs. Its inclusion in the spectrum of mental health service delivery options worldwide deserves serious consideration.

For research and academia, the research would contribute valuable data and insights to the growing body of literature on SST, particularly in the context of India and its cultural nuances. The BAT-Q and COSE-TR will be validated with Indian population and will be available for future use by researchers in India. Universities or training institutes can onboard this training program as a certified course. It will inform future research directions like client outcomes, long-term effects, and specific population groups who might benefit the most from single session thinking.

## Limitations

While this research holds promising potential to address the mental health burden in India through SST training, it is important to acknowledge its limitations. Firstly, cultural nuances can be multifaceted, and the initial tailoring might not encompass all regional sensitivities requiring ongoing refinement. Secondly, relying solely on self-reported measures of attitude and self-efficacy carries the risk of bias. The participants may also vary in their initial training, experience, and exposure. Thirdly, the focus is on immediate changes in attitudes and beliefs, short term or long term follow ups might be beneficial. Finally, the web-based format, though accessible, might exclude individuals with limited internet access or technological literacy. Future research could explore long-term program effects, address access barriers, and incorporate extensive qualitative methods for deeper understanding.

## Supplementary Information

Below is the link to the electronic supplementary material.

Supplementary Material 1 (PDF 240 KB)

## Data Availability

The training program proposed in this study is adapted from the training and implementation plan described by The Bouverie Centre, La Trobe University, Australia available at https://doi.org/10.1002/anzf.1426 and described in the manuscript. No datasets were generated or analysed during the current study.
